# Building a data sharing model for global genomic research

**DOI:** 10.1186/s13059-014-0430-2

**Published:** 2014-08-11

**Authors:** Patricia Kosseim, Edward S Dove, Carman Baggaley, Eric M Meslin, Fred H Cate, Jane Kaye, Jennifer R Harris, Bartha M Knoppers

**Affiliations:** Office of the Privacy Commissioner of Canada, Ottawa, Ontario K1A 1H3 Canada; Centre of Genomics and Policy, McGill University, Montreal, Quebec H3A 0G1 Canada; IU Center for Bioethics, Indiana University, Indianapolis, IN 46202 USA; Center for Law, Ethics, and Applied Research in Health Information, Bloomington, IN 47408 USA; Maurer School of Law, Indiana University, Bloomington, IN 47405 USA; HeLEX-Centre for Health, Law and Emerging Technologies, University of Oxford, Old Road Campus, Oxford, OX3 7LF UK; Division of Epidemiology, Department of Genes and Environment, Norwegian Institute of Public Health, PO Box 4404, Nydalen, Oslo 0403 Norway

## Abstract

Data sharing models designed to facilitate global business provide insights for improving transborder genomic data sharing. We argue that a flexible, externally endorsed, multilateral arrangement, combined with an objective third-party assurance mechanism, can effectively balance privacy with the need to share genomic data globally.

## The opportunities presented by data sharing models

One of the great opportunities in the genomics era is exploring how human genes influence health, disease and biologic pathways, and how the knowledge gained can contribute to better health through both prevention and therapy. Researchers collaborating globally can gather sufficiently granular data to discover gene-environment-disease correlations for translational research and clinical application. Conducting scalable projects has been aided by the convergence of two key developments: vast improvements in, and access to, low-cost sequencing technology, and the increased power and sophistication of data analytics, driven by what has become termed ‘Big Data’ [1]. Big Data provides a new generation of data analytics technologies that extract value from large, complex datasets (including genome and health-related datasets) so as to enable rapid capture, discovery and analysis [2].

The analysis, integration and translation of these diverse types of health data present a real challenge for science and policy. Progress in our ability to impact human health is highly reliant on bringing genomic technologies to bear on Big Data in ways that maximize data use, while minimizing duplicative effort and costs. But leveraging such opportunities is contingent upon cultural and policy changes aimed at enhancing genomic data sharing across borders.

Data sharing and research collaboration have become increasingly pervasive in the genomic research community. Moreover, funders increasingly require researchers to have data sharing plans described in grant applications [3]. Propelled by the groundbreaking data release policy of the human genome project (HGP), known as the ‘Bermuda Principles’ [4], data sharing is now emerging in clinical research as well [4]. The genomic research community has further fostered a culture of collaborative data sharing through international research consortia and public research platforms [5,6]. These are built on the belief that combining and sharing datasets will generate the statistical power needed to accelerate discovery and translate research findings into clinical practice. Also driving such collaborations are public funding requirements to enable sharing and secondary analyses of data and the corresponding ethical obligation to share knowledge for the benefit of society [7,8].

While a culture of global research collaboration is emerging, significant policy impediments to transborder data sharing remain [9]. Given the growing interest to combine individual-level genotype and phenotype data to understand better the determinants of health and disease, the more realistic starting assumption is that such data are, or might be, personal in nature. Genomic and clinical data sharing as a practice is challenged by regulatory systems originally developed to protect personal data within single jurisdictions [10]. These older data protection regimes are no longer attuned to the evolving paradigm of large-scale global health research, often resulting in inefficient data flow, significant costs and delays. For instance, in a recent literature review cataloguing barriers to sharing in biobanks, Colledge and colleagues remarked that ‘the divergence of regulations on the … transfer … of tissues and data is repeatedly mentioned as an obstacle to international collaboration’ [11]. Although some jurisdictions legally permit the export of personal information for research purposes, many others still do not, making inter-jurisdictional data exchange between research collaborators difficult, if not impossible, to achieve.

Realizing the promise of Big Data to accelerate scientific discovery and improve global health is of paramount importance. So too is the need to respect personal privacy and preserve public trust in health research [12]. Reconciling data protection laws designed to restrict transborder data flows with the scientific needs to share data globally is the challenge.

Scientists are not the first to face this challenge. For over two decades, businesses driven by global competitive forces have sought to derive value from personal data and capitalize on this new form of international currency [13]. Several models have emerged for sharing customer and employee data between companies, subsidiaries, affiliates, data processors and other organizations in different countries [14]. While none of these models has been entirely successful, their implementation to date offers valuable lessons for genomic researchers equally motivated to share genomic and clinical data across borders.

Here, we review six international data sharing models established largely to improve data flows in global commerce (summarized in Table 1). We then explain how useful insights can be drawn from each of the models to inform how genomic and clinical data sharing can be facilitated. We use the Global Alliance for Genomics and Health (GA4GH) as a case study to illustrate how an organization could apply the best elements of these models to the genomic research context. Our approach is inspired by the guiding work conducted in the Public Population Project in Genomics and Society (P3G) [15] and the vision of a newly formed international group of ethical, legal and social implications (ELSI) scholars (called ‘ELSI 2.0’) that, together, develop innovative tools and frameworks for enabling global, interdisciplinary genomic research in the public interest [16].

**Table 1:** **Advantages and disadvantages of six transborder data sharing models**

**Transborder data sharing models**	**Prototype**	**Description**	**Advantages**	**Disadvantages**
**1. Adequacy**	EU Directive 95/46/EC, Arts [25,26,30]	Personal data can be transferred to a foreign jurisdiction if its laws ensure an adequate level of protection in comparison with those of the exporting jurisdiction	• Ensures that the privacy laws of a foreign jurisdiction provide an adequate level of protection *before* personal data are sent to that foreign jurisdiction	• Long, slow process of adequacy designation - very few designations made to date
				• Imposes the privacy law regime of one jurisdiction on those of others
				• Only allows personal data to flow from a jurisdictional hub to the end of one spoke at a time
			• Provides upfront assurance and confidence on a country-wide basis	
				• Does not allow data-flows ‘around the wheel’
**2. Safe harbor**	The EU-US Safe Harbor	Personal data can be transferred to a non-adequate foreign jurisdiction (designated a ‘safe harbor’) if organizations in the safe harbor voluntarily self-certify that they will comply with mutually agreed-upon data protection principles (for example, access, security, data integrity, enforcement)	• Allows personal data transfers to a foreign jurisdiction without adequate legislation	• Lacks transparency and strong enforcement mechanisms
	Framework			
			• Administratively quite simple and well suited for small or medium-sized business entities	• No upfront assurance or third-party certification provided
				• Can only allow data to flow unidirectionally from one jurisdiction to another, not multidirectionally or between other countries
			• Organizations join based on a self-certification process - therefore, few resources needed to administer	
**3. Binding corporate rules (BCRs)**	Binding Corporate Rules of the EU	A multinational company can transfer personal data to affiliates and subsidiaries in foreign jurisdictions without adequacy status if it submits its global privacy policies and practices to a ‘lead’ data protection authority (DPA) for review and prior approval	• Allows data transfers to affiliated organizations in foreign countries without adequacy status	• Only applies to organizations within a single corporate entity
				• Process of review and approval is lengthy and bureaucratic
			• Allows data transfers to multiple countries at once	• Not well suited for small- or medium-sized organizations
				• Not easily scalable if many applications are submitted for approval to the same DPAs at once
			• Provides upfront assurance that BCRs will provide sufficient privacy protection	
**4. Model contracts**	EU-approved model contracts	Personal data can be transferred from one organization to another organization situated in a non-adequate foreign jurisdiction if the organizations agree to enter into a model contract pre-approved by the relevant DPA(s) as providing sufficient privacy protection	• Allows data transfers to organizations in countries that do not have adequacy status	• Of limited flexibility as model contracts must be used as they are, and any amendments must be resubmitted to the relevant DPAs for approval
				• Multiple contracts are required for data to flow to several organizations or countries
			• Provides upfront assurance that agreements are ‘up to snuff’ and will provide sufficient protection	
				• Not currently suitable for multidirectional/multiparty flows
			• Establishes grounds for contractual liability in the event of noncompliance	
**5. Accountability**	Canada’s PIPEDA, Principle 4.1.3	Organizations remain accountable for personal data in their possession and transferred to third-parties for processing. The transferring organization must use contractual or other means to ensure that the personal data continue to receive a comparable level of protection along the ‘chain’ of third-party transfers	• Ensures comparable-level protection along the entire chain of third-party data transfers	• No front-end assurance or certification provided
				• Requires transferring organization to carry out due diligence of third-parties and assume the risks related to data transfer
			• Transferring organization remains ultimately accountable	
			• Light, flexible, not front-loaded	• Weak enforcement mechanisms available if things go wrong
			• Focused on the ends of privacy protection, not the means	• Monitoring capabilities can be limited
**6. Third-party certification**	The APEC Cross-Border Privacy Rules Framework	Jurisdictions agree upon a series of privacy program requirements and third-party ‘accountability agents’ review and certify voluntarily participating organizations against those requirements. Once certified, an organization can partake in foreign data transfers, although still subject to the applicable privacy laws. Participating jurisdictions must have a domestic privacy regulator for enforcement purposes	• Provides upfront assurance, although less rigorous than DPA-approved BCRs	• Variation of laws remains a challenge
			• Could be flexible and expedient, depending on the efficiency of the accountability agent	• Little experience to date of how it works in practice
			• Scalable mechanism capable of handling large numbers of applications	• Currently, does not include EU countries, which remain subject to the foreign transfer limitations of the EU Directive
			• Does not displace domestic laws, but provides additional assurance that facilitates acceptance of foreign transfers	• There are some questions with respect to the rigor of the upfront assurance and the independence of the designated accountability agents

Six different data sharing models have been developed largely to improve data flows in global commerce: adequacy; safe harbor; binding corporate rules; model contracts; accountability; and third-party certification. Each model by itself possesses advantages and disadvantages. In this article, we argue that a new model, drawing from certain attributes of each, could be designed and adapted to facilitate global genomic and clinical data sharing in the Big Data era. *Abbreviations*: *APEC* Asia-Pacific Economic Cooperation, *BCR* Binding Corporate Rules, *DPA* data protection authority, *EU* European Union, *PIPEDA* *Personal Information Protection and Electronic Documents Act*.

## Adequacy

The European Union’s Data Protection Directive 95/46/EC (the ‘EU Directive’) generally prohibits exporting personal data of EU residents without consent unless the European Commission has determined *a priori* that the privacy laws of the importing country provide adequate protection [17]. To date, only a handful of non-EU countries, such as Argentina, Canada, Israel and New Zealand, have been granted ‘adequacy’ status under the EU Directive [18]. Adequacy in this context is a functional concept that means that the data protection regime of the importing country affords a sufficient level of protection, judged by both the intended data processing activity itself (for example, nature of the data, purpose and duration of the processing operation(s)) and the legal regime or measures applicable to the data recipient (for example, general and sectoral rules of law, professional requirements and security measures) [19].

The adequacy model provides strong upfront assurance that privacy will continue to be protected abroad, and, once a non-EU country obtains an adequacy designation, all data transfers from the EU to that non-EU country are permitted. As drawbacks, however, this model imposes the views of the ‘data export’ nation on other countries, the process for obtaining adequacy status can take many years, and, although the model allows free data flows between EU and adequate non-EU countries, it does not allow sharing with countries not recognized as adequate.

## Safe harbor

With no comprehensive data protection law, the USA does not meet the EU’s adequacy criteria. Given powerful commercial incentives to enable trade with the USA, an alternative arrangement, known as the ‘US-EU Safe Harbor Framework’ , was developed to allow the export of EU data to participating US companies, notwithstanding the lack of adequacy status conferred upon the USA [20]. To enter this ‘safe harbor’ , US organizations self-certify that they will comply with seven safe harbor principles. Adherence is enforced through the powers of the US Federal Trade Commission to investigate companies for false and misleading practices. To date, over 3,000 US companies have registered in the program [21]*.*

This model is straightforward to administer from a regulatory perspective, and entry into the safe harbor is based on a flexible, voluntary commitment of adherence. As a self-certification process, however, it does not provide the objective assurance of other models. Moreover, only a nation with sufficient economic and political clout can negotiate such an exceptional arrangement. That said, even nations as powerful as the USA are not beyond ongoing scrutiny. Indeed, in March 2014, the European Parliament backed a resolution calling for the suspension of the US-EU Safe Harbor Framework owing to concerns that it does not adequately protect European citizens [22]. Should the Framework be suspended, it will have a detrimental impact on organizations that crucially depend on data exchange between the EU and the USA.

## Binding corporate rules

Binding corporate rules (BCRs) are another exceptional means of exporting personal data outside the EU. Multinational corporations with pre-approved BCRs can transfer personal data within their corporate entity, including affiliates and subsidiaries in non-EU countries that do not possess adequacy status. A multinational seeking approval must submit its global policies and practices to a ‘lead’ EU data protection authority (DPA) - typically in the country of its European headquarters. Once the lead DPA gives its ‘stamp of approval’ , a mutual recognition scheme among most EU member states facilitates approval by other relevant DPAs [23]. To date, over 50 corporations have received BCR approval [24].

Approved BCRs provide upfront privacy assurance and allow data transfers between organizations in different jurisdictions, but only if they form part of the same corporate entity. From a regulatory perspective, the BCR approval process can be lengthy despite the mutual-recognition scheme and is not easily scalable to handle many applications at once. Although BCRs have only been approved for certain multinationals, the concept could, in theory, be applied to other entities, such as not-for-profit international research consortia.

## Model contracts

Model contracts are yet another mechanism created to permit the transfer of EU personal data to non-EU countries. The European Commission can pre-approve standard contractual clauses that build in sufficient protection for foreign transfers [25]. To date, the Commission has approved two sets of contractual clauses for the export of personal data outside the EU. Organizations wanting to use these pre-approved model clauses must use them as they are - any amendments must be submitted for approval by the relevant DPA.

Provided there are no amendments, these pre-approved model contracts can be a quicker, more cost-effective approach of providing upfront assurance by DPAs. Model contracts have the further advantage of permitting data transfers outside a single corporate entity, which BCRs do not allow. Although model contracts approved to date have been conceived as bilateral agreements, there is nothing preventing the possibility of having multilateral agreements between the multiple parties of a broader consortium pre-approved by the European Commission.

## Accountability

The accountability model is typified by Canada’s *Personal Information Protection and Electronic Documents Act* (PIPEDA) [26]. In contrast to the EU’s adequacy model, which assesses jurisdictional laws based on geography, PIPEDA focuses on the organizations involved, wherever situated, holding them accountable for the personal data they have, including personal data transferred to foreign third-parties for processing. The transferring organization must use contractual or other means to ensure that personal data continue to receive a comparable level of protection along the ‘chain’ of third-party transfers, but ultimately remains accountable for its weakest link.

PIPEDA does not require prior approval by the relevant DPA; instead, it provides an after-the-fact complaint mechanism for individuals seeking to challenge the level of protection. Arguably, this is among the most flexible models, but the limited regulatory scrutiny might be insufficient to secure public trust, particularly when dealing with sensitive health data. Although organizations with requisite bargaining power can dictate the privacy practices of contractors and subcontractors, those of smaller size and influence are less able to do so.

## Third-party certification

In 2011, the Asia-Pacific Economic Cooperation (APEC) economies endorsed a cross-border privacy rules (CBPR) system to facilitate data sharing in the Asia-Pacific region [27]. To participate, economies must have a domestic privacy regulator and at least one accountability agent (AA). AAs are third-party entities (either public or private) that review, assess and, if satisfied, certify the personal information management practices of an organization against a series of program requirements based on the APEC Privacy Framework. To date, three economies - the USA, Mexico and Japan - have been accepted, and Canada has announced its intention to participate [28]. Although experience with this model has been limited, the APEC Data Privacy Subgroup and experts from EU data protection authorities recently developed a practical tool (termed a ‘referential’) that compares the CBPR system with BCRs in an effort to facilitate inter-regional interoperability [29].

This model is flexible, scalable and provides upfront assurance. It potentially allows many participants to join, facilitating transborder data flows across multiple jurisdictions at once. However, reaching common agreement on framework rules, passing the rigorous upfront scrutiny needed to gain entry, and determining which bodies qualify as legitimate third-party certifiers can be challenging.

## Lessons for global genomic research

Given their respective limitations, none of these international data sharing models, developed largely to facilitate transborder data sharing in support of global business transactions, can be wholly transposed to the genomics research context. Yet, useful insights can be drawn from each of them to inform how genomic and clinical data sharing can be facilitated globally.

First, given the objective of accelerating statistically significant findings by combining, analyzing and comparing genomic data across as many researchers and research institutions around the world as possible, an inclusive multilateral arrangement would seem better suited than discrete bilateral arrangements.

Second, a scalable model could better accommodate increasing numbers of collaborating researchers and institutions wanting to join the data sharing arrangement over time, without bogging down the arrangement or imposing undue burden on the limited resources needed to regulate entry.

Third, given the sensitive nature of genomic and clinical data, providing upfront assurance before sharing data would be crucial for building and maintaining public trust [30]. External endorsement of the overarching data sharing arrangement by relevant data protection authorities or recognized third-party certification bodies would ensure that it meets different regulatory requirements and that researchers interested in joining comply with common principles or rules governing the arrangement.

Fourth, a trustworthy data sharing model for genomic and clinical data requires effective enforcement measures in cases of noncompliance [31]. These could include data access prevention, expulsion from the sharing arrangement and, in appropriate cases, investigation and possible sanction by relevant regulatory bodies.

Finally, internal ‘data user accountability’ [32] beyond mere legal compliance is needed to hold members of the arrangement accountable for how they use and manage data on an ongoing basis - especially in this era of Big Data, where it is nearly impossible to circumscribe the purposes for collection or limit future uses [33]. Members would be expected to assess potential harms and benefits, adopt effective safeguards for mitigating risks and implement robust governance processes for overseeing data access and use. Such governance processes have traditionally included policies, processes and oversight mechanisms, but increasingly also include: participant interfaces that give individuals greater control over their information, ‘e-governance’ systems that emphasize the use of technology to ensure compliance with ethical and legal requirements, and ‘adaptive governance’ systems that are responsive to changing conditions and allow for greater community engagement [12].

Given the considerations above, a data sharing model specially customized for global research consortia could well have the following traits: a flexible, multilateral arrangement, endorsed by relevant data protection authorities (such as BCRs or model contracts), and combined with an objective third-party assurance mechanism that regulates members’ entry and ongoing access (such as accountability agents in the APEC CBPR system). Until demonstrable evidence bears out the strengths and weaknesses of this and various other models (as has been proposed in other settings [34]), it is useful to consider how these suggestions could apply to a real-world case study, namely the newly established Global Alliance for Genomics and Health (GA4GH).

## A case study: the global alliance for genomics and health

In June 2013, a broad and diverse coalition of leading health and research organizations united with a global mission to accelerate progress in science and medicine through global data sharing. The GA4GH [35] was created as an international umbrella organization to develop and promulgate harmonized approaches (both technical and regulatory) for the effective and responsible sharing of genomic and clinical data across jurisdictions [36]. Currently, it has over 200 partners in more than 30 countries. The GA4GH seeks to work collaboratively with its membership to play an active role in catalyzing data sharing among members to advance science and improve human health. At the same time, it works together with its members to promote the highest standards for ethics and enable participant choice to share their genomic and clinical data responsibly and securely in order to contribute meaningfully to the advancement of human health.

International collaborations such as the GA4GH provide a timely opportunity for imagining a global data sharing arrangement based on some of the desirable traits canvassed above. To gain acceptance by the regulatory community and broader public, a data sharing arrangement between members of the GA4GH would have to be clear and transparent about its purpose: to improve global health in the public interest. As an overarching ‘consortium of consortia’ that includes both the for-profit and not-for-profit sector, the GA4GH would need to have flexible, multilateral arrangements in place. Whether centralized or federated, research initiatives using these GA4GH arrangements could then benefit from the prior formal endorsement by as many data protection authorities as possible. Entry into GA4GH projects could be subject to a scalable third-party certification process that assesses interested parties against commonly recognized principles and objective criteria. This upfront assurance could be complemented by internal accountability mechanisms for overseeing ongoing data access and use, reinforced by serious sanctions for noncompliance.

The GA4GH Regulatory and Ethics Working Group, of which several of this paper’s authors are members (BMK, ESD, EMM, JK), is actively implementing this vision of a flexible, multilateral arrangement by developing a ‘Framework for Responsible Sharing of Genomic and Health-Related Data’ (the ‘Framework’). The Framework is incorporated by reference into a constitution endorsed by GA4GH members. The Framework is founded on, and guided by, the human-rights principles of privacy, non-discrimination and procedural fairness [3]; it provides a principled and practical framework for the responsible sharing of genomic and health-related data between multiple international organizations, including the Public Population Project in Genomics and Society (P3G), the International Cancer Genome Consortium (ICGC), H3Africa, the Biobank Standardisation and Harmonisation for Research Excellence project (BioSHaRE) and the International Rare Disease Research Consortium (IRDiRC).

The Framework will be elaborated by subsequent policies on particular issues such as ethical governance, consent, privacy and security, and, in so doing, will elucidate the various core elements of responsible data sharing. The Framework and policies, particularly if endorsed by multiple data protection and research-ethics oversight-authorities across various jurisdictions, could be used in genomic research projects around the world, whether GA4GH-‘inspired’ or not. Recognizing diversity of legal and ethical approaches and being responsive to emerging issues, both the Framework and the policies can hopefully serve as a potential model and provide leadership in this domain for wider discussion. Through its international collaboration, sharing of best practices and cross-pollinating of ideas and learning, the GA4GH serves as a powerful case study of how the best elements from data sharing models developed largely for commercial purposes can be applied to the genomic research context.

## Concluding remarks

We believe that it is possible to protect privacy while also enabling societal benefits that come from the use of data. Just as Big Data is changing the way genomic science is conducted, so too is it changing the way it must be governed. We have discussed six transborder data sharing models largely stemming from a commercial context that might, when compared and combined, offer valuable lessons for genomic research collaborations. The fact that international data sharing models, however imperfect, were able to emerge when commercial incentives were sufficiently strong should offer hope for genomic researchers equally motivated and engaged to share data for even more socially valuable purposes.

## Abbreviations

AA: Accountability agent
BCR: Binding corporate rules
BioSHaRE: Biobank standardisation and harmonisation for research excellence project
CBPR: Cross border privacy rules
DPA: Data protection authority
ELSI: Ethical, legal and social issues
EU Directive: The European Union’s Data Protection Directive 95/46/EC
GA4GH: Global Alliance for Genomics and Health
HGP: Human genome project
ICGC: International Cancer Genome Consortium
IRDiRC: International Rare Disease Research Consortium
P3G: Public Population Project in Genomics and Society
PIPEDA: Personal information protection and electronic documents act
